# Narratives of heritage and legacy: Child and adolescent mental health trainees portrayed

**DOI:** 10.3389/frcha.2023.1104637

**Published:** 2023-02-23

**Authors:** Ayobello Ayotunde, Karen Siegel, Adelaide Feibel, Laelia Benoit, Andrés Martin

**Affiliations:** Child Study Center, Yale School of Medicine, New Haven, CT, United States

**Keywords:** child mental health, professional identity, qualitative methods, narrative analysis, art and medicine, heritage and legacy

## Abstract

**Objectives:**

We sought to embellish a child and adolescent mental health space by creating and displaying a large painting imbued with meaning and symbolism specific to the field. In it, we featured a broad array of trainees in the disciplines of child psychiatry, psychology, and social work. We used the portraiture sessions as opportunities for participants to reflect on their professional trajectories and developmental progression.

**Methods:**

The lead author painted *Heritage and Legacy*, a 6 × 4 ft oil painting of 15 trainees (8 women), between February 2020 and December 2022. Each studio sitting lasted approximately four hours and included an in-depth interview that was recorded and transcribed for qualitative analysis. We used narrative inquiry as our analytic approach, a method that attends to unique stories and aims to make meaning out of individuals' life experiences.

**Results:**

We organized our analytic framework chronologically: (1) *Heritage* (past influences); (2) *Becoming* (the current process of professionalization); and (3) *Legacy* (reflections about the future). Through these life stages, we consider findings from three complementary vantage points: (1) the unique methodology of using a collective portrait as the basis for a qualitative study using narrative inquiry; (2) the participants' individual and collective trajectories of professionalization and professional identity formation; and (3) their transitions and legacy—including through mentorship and generativity, as much as through concrete objects and places, such as the oil painting itself and the space in which it will permanently reside.

**Conclusions:**

The lengthy process of creating an oil canvas depicting a multidisciplinary group of professionals in training proved a powerful vehicle for self-reflection by those portrayed. It has yielded insights of broader relevance to the training and education of the next generation of practitioners.

We do not only define ourselves; we are also defined by our circumstances, culture, the perceptions of others and—perhaps most of all—the force of an internalized past.

—Eva Hoffman, 2004 (1, p. 27)

## 1. Introduction

Portraiture is one way for institutions to display, preserve, and share their values in a lasting manner. Medical schools, hospitals, and other medical spaces worldwide have embraced this type of visual representation. Medical iconography has been defined as the study and “enumeration of *outstanding physicians* through our recording of their portraits” (2, emphasis added). This long tradition has recently come under scrutiny and poses important questions. Among them: should it only be “physicians” who are portrayed? Or should the focus be exclusively on those who are “outstanding”? More to the point: with the vast majority of extant paintings portraying men—white men at that—what is the implicit messaging on display?

A qualitative study (3) documented the impact on medical students traversing hallways decorated with such portraits. For many, an eroded sense of belonging to the institution resonated in at least one of four ways: (1) Institutional values (e.g., “Who does my school value, or doesn’t?”); (2) Resignation and coping (e.g., “If this has been so for centuries, it will remain the status quo.”); (3) Contemporary consequences (e.g., “I'm not welcome here.”); and (4) Erasure of history (e.g., “The contributions by women and people of color are overlooked and not as valued.”). A feeling of alienation from one's own workspace, reinforced by a mismatch with the racial homogeneity of such portraiture has even contributed to some physicians of color leaving the profession altogether (4).

Two ways of addressing these concerns are being tried at different institutions: a “dispersive” approach, and an “additive” one. In the former, first implemented at Brigham and Women's Hospital in Boston (5), the portraits of physician leaders (virtually all of them white males) were relocated to alternative and less prominent parts of the hospital, making space for fresh and more inclusive imagery. In the latter approach, new portraits are commissioned with diversity and broad representation as core requirements (6). A hypothetical question may be best posed in the reverse: if lack of identification can result in alienation and a diminished sense of belonging for colleagues from underrepresented minorities, what could be the impact of more concordant portraiture? One pithy response is on point: “seeing role models look like us in spaces we would like to occupy is powerful: our imaginations begin to position us in the places of those we revere” (7).

By virtue of their transient educational experience in an institution, trainees seldom have been represented in medical iconography. A telling exception is *Charcot's Clinical Lesson at the Salpêtrière* (8), a classic painting that does include trainees (among them a young Charles Gilles de la Tourette and Sigmund Freud), but who are nevertheless depicted as fawning background pieces rather than as protagonists in their own right. The omission in medical portraiture of medical students, residents, fellows, and others in training represents missed opportunities. First, that of putting on display the values and priorities of an institution, including its embracing diversity and belonging, valuing the educational mission, and caring for the developmental progression of those most directly served by it. Second, and related, prominently displayed visual signifiers such as a permanent oil painting can have an impact in fostering recruitment and solidifying retention efforts of trainees and faculty.

With these considerations in mind, we undertook a project consisting of three interrelated goals: (1) To embellish a child and adolescent mental health center by creating and displaying a large oil portrait imbued with meaning and symbolism specific to the field; (2) To feature in the painting the broad diversity of the center's trainees, and specifically including different professional disciplines (child psychiatry, psychology, and social work); and (3) Given that portraits invite reflection not just in the viewers, but in its subjects as well, we used interviews during the portraiture sessions as opportunities for the trainees to reflect on their trajectories of professional development. Considering that the painting itself would become a legacy gift to the center, a question embedded from the start was for trainees to share their notions and feelings around the word itself—legacy.

## 2. Methods

### 2.1. Portrait sitting and interviews

We invited trainees in the disciplines of child and adolescent psychiatry (CAP), psychology, and social work at the Yale Child Study Center (YCSC) to be depicted in a permanently displayed oil painting. Volunteers agreed to be interviewed and audio recorded as part of their sitting. They each provided informed consent and knew their de-identified comments would not be linked to their facial likenesses. There were no hierarchical or power-related concerns in participating or passing on the opportunity given that no one with supervisory or evaluative oversight was involved in the invitation, consent, or interview process. Each studio sitting lasted approximately four hours, with half of the time dedicated to an interview that was recorded and later transcribed. The lead author started painting in April 2020, continued as the sittings and interviews progressed, and completed the canvas in December 2022. The study was approved by the Yale Human Investigations Committee (protocol #2000031551).

The 6 × 4 ft (1.83 × 1.22 m) canvas was painted by the lead author, at the time a child and adolescent psychiatry fellow at the YCSC. A self-taught oil painter relying on the “thick over thin” method toward a self-described “psychiatric surrealist” style, he had previously worked on topics at the intersection of art and mental health, such as perinatal depression (9). He led the interview of each participant—his fellow colleagues-in-training—during allocated time within each of the sittings. A different team member interviewed him in turn, since he, too, was portrayed in the canvas. The in-depth interviews aimed to elicit the personal and professional influences leading to the present moment in each of the participants' lives. With trainees fast approaching their graduation, the interviews were also centered on anticipated plans ahead, both for the near term and the future. In preparation for the sitting, we encouraged participants to bring anything (or anyone) personally meaningful to the discussion, or who would ease their experience. Interviews were free-flowing and loosely incorporated some of the sensitizing questions listed in Supplementary Appendix S1.

The painting can be conceptualized as an “intervention” and the related interviews as data collected toward a study about the self-reflection and professional development of a group of child and adolescent mental health professionals-in-training.

### 2.2. Qualitative analysis: narrative inquiry

We recorded digitally all interviews, transcribed them verbatim using Rev (rev.com), and uploaded them into NVivo (MSR International, Melbourne) toward qualitative analysis. We used narrative inquiry (alternatively, narrative analysis, NA) as our analytic approach. The focus of NA is on individuals as the unit of analysis; it attends to the unique “voices” underlying their speech, writing, or thought, and aims to make meaning out of life experiences (10, 11). With this attention to detail and specificity, narrative inquiry seeks “to preserve the vitality of individual voices” (10).

Medical socialization is a process through which students learn how to become doctors (12). In the context of this project, we consider the term “medicine” broadly, and use it interchangeably to include other pediatric mental health disciplines. We informed our analysis with an interactionist perspective (13) on medical socialization: interactionism states that individuals learn to play social roles through repetitive interactions with others. As such, learning the culture of medicine, its ethos, occurs by the repetitive socialization of certain narratives. By “ethos” we mean the fundamental character or spirit of a culture, era, or community—as manifested in its beliefs, customs, practices, and aspirations. To assess the ethos of participants, we analyze not only what participants share about themselves (narrativity), but also their discourse on the culture, values, and beliefs of their professional group (socialization).

Two of the senior authors are experienced qualitative researchers (LB, AM). One of the team members served as primary coder; two others as secondary. During periodic meetings, they resolved questions, triangulated findings, determined data sufficiency (14), and arrived at a joint codebook. The final codebook informed an analytic framework organized into domains, themes, subthemes, and supporting quotations. We followed best practices in the reporting results of qualitative research (15).

### 2.3. Reflexivity

The lead author, as documented in his self-portrait within the painting, is part of the group he has set out to paint and interview. As a CAP approaching the end of his own training, he is leading a research effort of a group of professionals looking at themselves. The other authors are currently, or intend to be, in the field of CAP, further highlighting the project's “self-reflective” perspective. The collegial relationships between the “insider” artist and his peers likely contributed to rapport and comfort during their one-on-one sessions. Notably, as a Black man, his stance regarding where the painting would reside (in a traditionally white medical space), is intentional and an important driver of the introspection, intersubjective reflection, mutual collaboration, and social critique underlying the project (16).

## 3. Results

Among the 26 graduating trainees in the 2021 and 2022 cohorts, 16 (62%) sat for a portrait. The majority were from the child psychiatry program (11/14, 79%), followed by social work (2/4, 50%) and psychology (2/8, 25%). Nine of the trainees were women (56%), and four psychiatry trainees were international medical graduates (36%).

**Figure 1:**
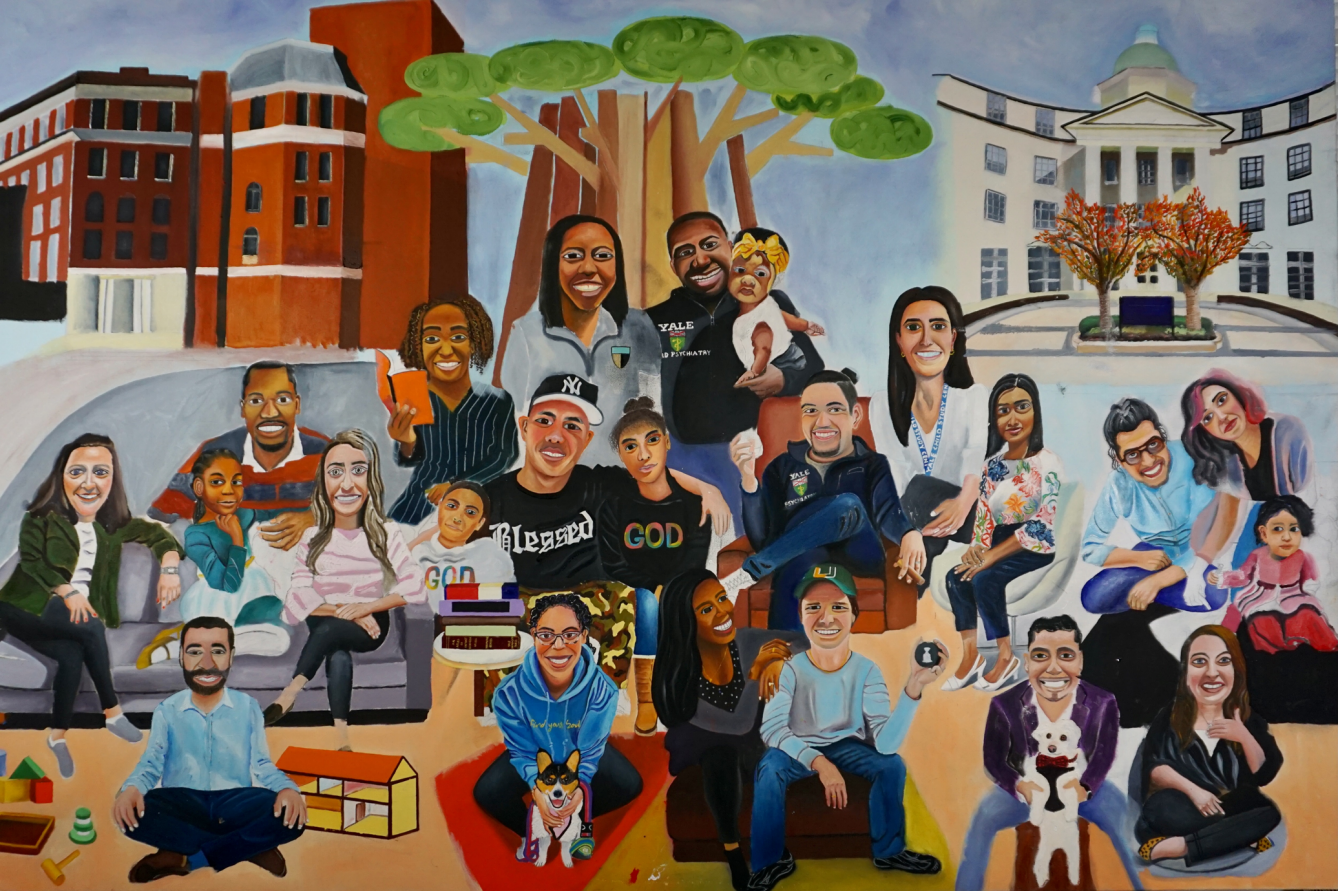
*Heritage and Legacy*, oil on canvas by Ayobello Ayotunde, 2023.

The finished painting, *Heritage and Legacy* (Figure 1) portrays 23 individuals: 15 trainees and three partners. It includes five underage offspring of the participants: an infant, a toddler, two school age girls, and a young adolescent. A doll's house and wooden blocks on the lower left represent *play as a tool of healing.* That tradition resonates in the adjacent spines of canonical textbooks in child and adolescent mental health, including those by Leo Kanner, Arnold Gesell, Melvin Lewis, Albert Solnit, and James Comer (all members of the YCSC, except for Kanner). The young professionals' diversity in display is complemented by the Pride flag and a pin with the transgender colors. The academic and interpersonal nourishment during the years of training is symbolized at center left by meals at the street food carts near the Medical School, and the threats and challenges unique to 2019–2022 are noted by markers of the COVID-19 pandemic. The group of trainees and their children are loosely organized in a pyramidal shape whose central apex merges with a tree trunk and its overhanging canopy. The tree—a universal symbol of generativity—is also an accurate depiction of a wooden sculpture at the YCSC's outpatient clinic's waiting room. As the Center's newest building, the outpatient clinic and its iconic tree ground the training experience and provide a sense of place. As a metaphor, the tree's roots connect to the past, to a source depicted by other buildings of the YCSC (upper left) and the Yale School of Medicine (upper right). As a final metaphor, the trees' roots stand for age and depth—for heritage—and its limbs, leaves, and green shoots, for youth, growth, risk, and challenge—for legacy.

We approached the corpus of the participants' individual interviews using narrative analysis. We organized the resulting domains chronologically: from past influences (heritage), through the current process of professionalization (becoming), to reflections about the future (legacy; Table 1). We go on to describe each of these three epochs of professional development and reflection.

**Table 1:** Analytic framework.

Domain	Theme	Illustrative quote
**Heritage** (the past)	Appreciating family and community	Because of having older siblings, I feel that in school my grades were good because they always tutored me, they always gave me good advice. I also think it's easier to be a younger child because you get so much experience from your older siblings.
	Remembering challenges and hardships	[My father is] an amazing human being, but he didn't read the book on parenting. He didn't have that gift.
	Incorporating experience toward building more resilience	I'm doing the work. I feel good. I feel strong. I haven't had all of those things that have been occurring to my family for so long. Like “be proud of yourself for that,” and just continue. I'm happy with where I am right now because I am showing up every day.
**Becoming** (the present)	Transitioning from trainee to professional	A healthy dose of imposter syndrome keeps me motivated. I don't know if there's a healthy dose of this, but…a healthy dose that I'm not good enough, keeps me on my toes and keeps me reading, and keeps me studying and keeps me improving.
	Belonging to a field	I really felt for the first time a sense of community and a sense of belonging. And it has given me a great sense of confidence.
	Embracing one's full self	I knew at that point that I was psychologically minded. And I also knew that I loved learning about religion and philosophy, and I wanted to somehow combine those two and I thought, what better place to combine them than in psychiatry?
**Legacy** (the future)	Envisioning what is left behind	The reverberations that the past is having on the present, especially from certain individuals or groups or actors or forces or whatever they are, the way they are influencing the now and the way they (and perhaps we) will continue to influence the future. The person who is the receiver has a memory of what you give; that thing is being passed on: legacy is a memory passed between people. If heritage is something I’ve been given, legacy is something I give.

### 3.1. Heritage (the past)

In reflecting about their pasts and the influence of their upbringing and trajectory up to the present moment, participants' comments fell into one of three broad categories:

#### 3.1.1. Appreciating family and community

Family relationships were at the core, reported by virtually everyone as the primary influence on their lives, education, and career decisions. Heritage was described as a palpable gift, as something concrete and useful to be bequeathed:

I will leave you no inheritance other than your education.

Much like age, the sitting sessions and the interviews provided an opportunity to appreciate the difficult choices their parents and families may have had to make along the way:

…as I get older, I appreciate the sacrifice, which makes all those memories even more beautiful.

Different parenting styles came into focus, some of the “tough love” kind, others driven by guilt and externalities, yet others providing templates for either apprehension or encouragement:

My dad was like, “Don't climb that tree.” My mom's like, “How high can you go?”

Friends, sports, faith, mentors, school, and community relationships were commonly mentioned as helpful to eventual professional development, as in one example of the support of peers when facing a threatening encounter:

We were still part of the same projects. And that was a shared identity, that we would have each other's back. But we had a thing, a saying, “We're from down the hill, never ran, never will.” And that stuck to me, because it created this sense of determination and grit that I'm never going to run away from anything. I was facing this head on and would not stop until I got my goal.

#### 3.1.2. Remembering challenges and hardships

Some participants were aware of having grown up in less-than-ideal conditions, with insufficient structure, even without preconditions for basic safety. Living in large urban areas made such shortcomings particularly apparent. Being at odds with parenting styles or family priorities on occasion had a direct impact on education and long-term career goals:

Those were never my values. I only realized it after high school because my parents spent all their money on flashy things, but then had to take out loans for me to go to college.

Ruptures and division in families were not just around values: divorce or death led to major derailments for some. And yet, the experience of loss could also prove useful in understanding others:

People are often thinking about all of the memories that have happened in the past and grieving those memories. Whereas, for me, and what I found for some of the families I work with, it's more grieving the future that you could have had. Yeah, I think my grief was more future-oriented than past-.

Physicians in the group reported unique challenges. Family pressure to pursue medicine as a socially accepted and prized profession could be alternatively uplifting and stifling. Disappointment with medical school could be hard to acknowledge to loved ones, as when struggling to actualize cherished aspects of oneself during the lengthy years of training:

I was calling my mom and telling her “I'm not happy. I don't think I want to be in med school. I want to do something creative. I don't feel I'm alive.”

International medical graduates had to deal with additional hurdles: learning a new language (including the language of American medicine), passing equivalence exams, navigating new systems of care, or filing immigration and licensing paperwork. But the challenges of immigration and acculturation were not unique to physicians. Several participants commented on making their way into systems that had at times been unwelcoming, racist, sexist, or classist. Many recalled the initial narcissistic injury, or the lingering residue, of failing exams and other professional milestones:

It is hard to stay committed and to not completely let it just wreck you. Which when I didn't match on my internship the first time, it wrecked me for a couple of months, and I was down and did not want to study and didn't want to do a lot of things.

A prevailing myth of the perceived ease of others could only aggravate the difficulty inherent to professional development and progression:

You don't hear people's failures. You hear about how great they're doing and how things are going well for them.

#### 3.1.3. Incorporating experience toward becoming more resilient

Common to all participants was a tenacity to remain engaged in long-term study and work toward a planned professional goal. Trajectories were not necessarily linear or foreseen from the outset. Indeed, unexpected encounters were often transformative, more a matter of serendipity than of crude luck:

My career path was wide and zig zagging. But I think the common theme from all those experiences was at least one or two meaningful professional or personal relationships, where if I didn't take the job or if I didn't do the thing, then I would have never met the person.

Growth-enhancing experiences included taking advantage of available opportunities, as much as successfully navigating personal and familial challenges—including those of mental illness. Recovering from illness and contextualizing the experience toward a career dedicated to mental health was viewed by one participant as akin to overcoming adversity and learning to persevere in its wake:

After what I have gone through and overcome, I now have to try things even if I fall down: falling has taught me how to bounce back up again.

Adversity energized and propelled another's career, giving it direction and a sense of mission, of paying it forward:

You're going to somewhere better, and then you'll take this stuff and bring it back, and you're leaving a family unit for some time and you're going to create a better family unit. And it made me a better person because gratitude is a virtue. So, I look at my upbringing not as an obstacle, but as a badge of honor.

Letting go of painful memories in service of embracing new opportunities resounded with participants who had immigrated, or who were first-generation children of immigrants. For them, striving, progress, and acculturation inevitably meant grieving some of the cultural aspects of their countries of origin:

Because heritage doesn't necessarily last forever. Heritage may die with the people.

In becoming parents, some participants were keenly aware of how much—or how little—of their cultural heritage or native language they would be able to transmit, what would be feasible given the realities of growing up in a different predominant culture.

### 3.2. Becoming (the present)

Participants were approaching the end of their formal years of training at the time of sitting for their portrait and interview sessions. The timing was propitious for them to reflect on their development from the stage of trainee into that of professional, and on those aspects of the work—and in themselves—that first drew them in and kept them meaningfully connected to it.

#### 3.2.1. Transitioning from trainee to professional

Trainees were evenly split between those who had long wanted to pursue a mental health profession and those for whom it was an unexpected finding along their path. Among the former, personal or family experiences with mental health treatment were common—as was the impact of undiagnosed and untreated illness and an ensuing desire to make things better for others. For those in the unexpected pathway, the unique nature of mental health care of children and families stood out, particularly among the physicians in the group: interpersonal closeness and the ability to interact meaningfully with patients and families did not initially align with the traditional view of medicine they had been socialized under:

You just get to really listen and talk to people, and really get to know them, which is something so special that we don't get in a lot of other fields [of medicine], or other aspects of our life for that matter. That's what sold me.

The outsized role of psychosocial adversity in the daily clinical work could become overwhelming. It could also prove transformative:

Most of my patients live below poverty and the resources are not good for mental health. And then there is the stigma, the racism. Residency changed me as a person, my perspective, my point of view. That's the time I really got to see people, real people, their struggles, their suffering.

As they gained knowledge, skills, and became more facile in their interactions and interventions, they also started to harbor self-doubts and insecurities about their competence, about their impending autonomy:

Do I have anything to offer at this point?

They were not alone in seeking an endorsement or concrete validation of their hard-earned professional abilities:

It's ten years of training and it's all coming to an end. We never stop learning, but the protections, the cocoon, having your colleagues right next to you…It's all going to be different.

Participants repeatedly highlighted the role of mentors in supporting their development up to the present moment and making their upcoming transitions less daunting. More than any given piece of advice, or the instilling of unique wisdom or values, mentors were cherished as unique given their ability to see beyond each trainee's individual horizon:

I will still check in with them from time to time. I think they both saw in me from an early time something that I did not see in myself, and that I still question sometimes.

#### 3.2.2. Belonging to a field

Participants reported a range of motivators for pursuing their respective field and embracing its core values. For example, some were attracted by the scientific promise, while others were attracted by the flexibility of practice that could be possible upon graduation. But all had in common a commitment to help improve human relationships and attachments:

The relationship *is* the focus: creating safety in the relationship, overcoming trauma or challenges–*through* the relationship.

Relationships were referred to not only as instruments for healing, but as reaffirming a sense of purpose in the field of their choice. Clinical relationships provided long-term nurturance as well:

I, and they, are living these lives that I don't think we would have been living if we hadn't been able to work together.

Connecting to children and families through play or words provided opportunities to get ever closer approximations to core issues. In doing so, clinicians were better equipped to make a difference, and to redouble their sense of belonging to the field, as of connection to those under their care:

Being able to tell stories really connects me with my patients. Everybody has a story. Now that I see my patients, I really try to pay attention to the context of what's going on with them: what is the *heart* of the story?

Many of the narratives and complex dynamics that emerged in these exchanges were challenging. The ability to listen to traumatic histories involving minors had led others to abandon or not pursue the field in the first place. Alternatively, participants were often energized by working in the area of trauma, both by the help they were able to provide, as by gaining a sense of empathy for families affected by domestic violence or intergenerational trauma:

The people who are closest to you can also be the ones who have done all the harm. Coming into this field has softened me toward family and friends—mine included—and provided a different frame of understanding.

Several were drawn to fields in the pediatric mental health professions knowing them to be inherently collaborative and multidisciplinary, where they could overcome vicarious trauma,

by relying on colleagues and supervisors who I trust to deal with the heaviness of the work.

They valued their collaboration with schools, pediatricians, state agencies, and protective service, among others. The team-based approach usually started at the first visit, with the home “team” of the family, whether biological, adoptive, or foster, or with those taking care of the child in an alternative or congregate care setting. Sharing the responsibility of care and relying on complementary areas of expertise made the challenges of such complex care manageable rather than burdensome.

Yet burdens participants did share, including expressing dissatisfaction with a culture of metrics and quantification, an aspect of care in the current healthcare environment that can erode a sense of clinical agency:

There's a competition in general, how we have to compete for jobs, compete for…I hate this word: *clients*. This aspect of customer service, of following the results of satisfaction surveys, of electronic medical record metrics. “Are they happy customers?” Not “are we doing the right thing for the patient? Is this what they need?” Who is driving care these days?

#### 3.2.3. Embracing one's full self

The ability to remain playful, imaginative, and creative while doing the serious work of child mental health was a lasting draw for many participants. It connected them to their own experiences as children, it made the work lighter, even bearable at times. It also kept them intellectually engaged and honest. Questions or frames of reference that would have been construed as “unusual” or “unconventional” in the medical context in which the physicians in the group were educated, could become useful in the context of child psychiatry. In encouraging a medical student to be surprised by their own questions, a participant noted how

child psychiatry is the only place where if you ask off-the-wall questions, you might actually get off-the-wall answers that will surprise and help you. So, no bad questions in child psychiatry.

Long-standing avocations or interests were reported as essential to some of the participants: informing their practice, giving them specific skills, synergizing their more traditional “toolkit”:

I knew that I wanted to do psychiatry. I knew at that point that I was psychologically minded and that I would pursue it. But I also knew I loved studying humanities and wanted to somehow combine those two areas and I thought, what better place to combine them than psychiatry?

Left unchecked, self-reflection can devolve into solipsism. But when well tempered, particularly in the service of thinking about relationships and of others, as in the case of psychotherapy, reflexivity can be an effective tool. In becoming mental health professionals, participants embraced and refined their introspective and intersubjective reflective skills, given that

as child mental health clinicians, we are so used to focusing on the other person, the person in the chair. But I think it's useful to also have some personal introspection.

An honest, hard look at oneself can lead to difficult realizations. In their own words, two participants made an entreaty not to shy away from sharing some of those areas of vulnerability, but rather to use them judiciously toward the improvement of patients—and colleagues:

If we are able to share things that we have been through or share our lived experiences, even if they were difficult or hard or traumatic, and if we are able to tell our stories, I think it enables other people to be less alienated and alone.

You find these patterns in yourself. Just, you can't run from them. And you have to work through them.

### 3.3. Legacy (the future)

As they approached graduation and the end of their formal professional training, participants gave considerable thought to their futures. Not just about the immediate futures at their soon-to-start postings, but about their hopes and fears in the longer term. A wish to make an impact, to lead to changing children's lives for the better, was shared by all. This yearning to make a mark, for one's work to have lasting value and not fade away, was reflected in three quotes using the term “print” in efforts to define the term “legacy”:

It's your finger*print* on the world.

It's your energetic foot*print*.

What will be left when we are gone. The kind of im*print* you have on the world moving forward, because we all have an imprint of some shape.

Being “gone” in this context did not refer necessarily—perhaps not at all—to mortality. With most participants set to relocate, and with all of them about to have a major transition in their professional lives, the finality of their training invited reflection on other forms of legacy. What would they take from their years of training? What would they hope to pass on?

Becoming teachers, mentors, and sponsors to younger colleagues was high on the list for many. Passing on some of the gifts they had in turn received, finding their personal style to pay it forward were strong motivators to look forward to concretizing. Nor did becoming an effective mentor necessarily imply an arduous apprenticeship, because

you really don't know who you are influencing. People are watching you regardless of where you are and what you're doing. And so, we can have profound impact every single day—by just showing up.

Being uncertain about the specific influence on others was particularly relevant in a field dealing with children. Even if never learning the long-term outcome of many of those under their care, the group as a whole had a strong sense of mission and trust in their actions supporting youth and their families, helping ease suffering, or contributing to disrupt cycles of intergenerational trauma. They were keen on applying their professional training and individual skills toward a more equitable and socially just world. They were eager to increase the knowledge base of their field whenever possible. At the cusp of graduating, several participants were at once excited and apprehensive about the positions of leadership they would be starting imminently:

it's a pressure that I have kind of accepted and look forward to. Still, I am worried about…We don't get really a lot of leadership training. You get some, but not a lot. So, I'm also worried about, this is a space that is novel for all of us, at least in the beginning. But if I don't step into this role, who will?

The painting itself provided one final aspect for reflections on legacy—as a concrete representation of the past living on into the future. Participants saw the painting develop over time; they were aware of where it would go on to hang in their training institution,

a material legacy left behind; something beautiful left for everyone to see.

Just as they would not know the fate of many of their young patients many years later, participants were not sure what specific message would be conveyed through the oil canvas years hence. But in both instances, patients and painting, they had confidence in the value of the work and the positive ripples it would lead to over time:

You made the space safe; it feels like you're creating a legacy project. And that means that you are sending love out there, and it will stand the test of time. People will look back at these images and these interviews and gain something, be influenced somehow.

## 4. Discussion

We based a study about the life trajectories of trainees in the child and adolescent mental health professions on a large oil painting depicting their likenesses. We go on to discuss our findings from three complementary vantage points: (1) the unique methodology of using a collective portrait as the basis for a qualitative study using narrative inquiry; (2) the participants' individual and collective trajectories of professionalization; and (3) their transitions and legacy—including through concrete objects and places, such as the oil painting and the space in which it will permanently reside.

### 4.1. Strengthening reflective practice through a collective portrait

Sitting for a portrait is a deliberate undertaking. It involves openness, candor, and honesty; it can place the subject in a position of vulnerability. Alternatively, a sitting can make subjects feel truly seen, appreciated, and welcome. To accomplish such an environment, we encouraged participants to make intentional choices about their desired pose, and about the clothes, objects, animals, or persons they would surround themselves with. As the painting developed, they also came to see themselves surrounded by their peers: The first participant saw a large blank canvas with their lonesome portrait; a very different perspective from that seen by their subsequent peers, who came to find each other within a growing community. This dynamic evolution contributed to a component of collective meaning-making (17) that suffused the overall project, the shared initiative of two graduating classes.

Engaging in reflective practice on a consistent basis is of paramount importance for all health care providers. Through regular reflection on their experiences, clinicians can become more competent, empathetic, and ethical (18). A systematic review examined the salutary connection between critical reflection and (1) comfort with learning in complex situations; (2) engagement in the learning process; and (3) deepening of professional values (19). There are several empirically-tested approaches to enhance reflective practice, including those that use narrative medicine elements (20–22), simulation (23), or the visual arts (17, 24), among others. Visual art instruction is routinely used by some medical school programs who partner with art museums and their docents. By enhancing “visual literacy,” such programs have documented improvements in outcomes ranging from clinical observation skills, cultural sensitivity, and burnout prevention.

Our project was informed by prior initiatives at the interface of the visual arts, the health sciences, and reflective practice, but with a key difference on the relation between the learner and the canvas. Specifically, we intended each participant not just to reflect on, but indeed *become part* of the canvas. This is a first in child psychiatry, and we are not aware of comparable projects in other areas of medicine. This unique approach was richly conducive to reflection; it was inviting of spontaneity and creativity, and it encouraged reflection on participants' different identities and timescales. Other exercises using narrative reflection exercises have traditionally focused on current phases of training or on individual career paths. By contrast, our interviews were rich in content about families, peer groups, and other social domains; their time horizons were lifelong. The sittings and their interviews yielded content that was rich in social and societal influences on the trainees' lives and prospects; they were conducive to confronting and embracing ambiguity (25).

Uncertainty and ambiguity are spaces that child mental health clinicians must often inhabit: developmental progress can be nonlinear, clinical outcomes be difficult to predict, and different treatments yield similar results. Such factors have an effect on who may choose fields that are by definition not categorical in their solutions. To the contrary, they are disciplines of chromatic gradations, such as those captured on the canvas. The gravity of psychosocial adversity affecting minors that clinicians often face also compounds coping with uncertainty, to various degrees an inevitability in all medical disciplines (26). *Functional optimism* is one way in which child mental health professionals prevail when facing what could otherwise be overwhelming prospects (27, 28). The bright and playful qualities of *Heritage and Legacy* and the figurative and naïve style of its likenesses reflect such “ritualized” optimism (28).

### 4.2. Career choice and professional identity formation

The narratives we distilled represent not only individual stories. Their commonalities embody parts of the ethos of the child and adolescent mental health fields. Hopefulness and optimism (29) may be more common among those working with children. Although this thought has not been empirically tested, the traits were certainly common across all participants, who had a positive outlook of the future and of their ability to make a difference. This sense of agency toward improvement was the most common reason given for choosing their professions. The ability to work with children, families, and communities in a holistic way was also mentioned often, as was the opportunity to incorporate other aspects of the patient's self—and indeed, of their own, as in examples of the humanities, religion, or sports avocations. Participants were split between those who *chose* their field (e.g., “I always knew”) and those who *were chosen* (e.g., “I never thought of it, but after ruling out all other fields, it became obvious.”) It is likely that the stigma so potentially affixed to the field of child mental health (30) may have contributed to “never thinking of it.” But perhaps more notable, and in keeping with the literature (31, 32), is how so many participants leaned into the stigma of mental illness as a reason to pursue the field in the first place, as reflected by their own experiences with psychopathology in their families or in themselves. Also consistent with others' findings, neither compensation, delayed income growth, or additional years of training featured prominently as impacting career choices (33).

In the mental health disciplines, and as reflected in the interviews, learning how to share about oneself is an important element of the transformational and socialization process. It involves the genuine intersection of personal and professional identities (34), the bringing in of one's “full self.” Language, narratives, and sharing are essential tools for mental health clinicians to assess their patient's inner worlds, framing and reframing a central component of all psychotherapies. Sharing is also a way for trainees to show their mentors and supervisors that they can muster insight and reflexivity. Participants shared openly about themselves, and in doing so, displayed their clinical skill. Learning how to speak about oneself, about one's past, family history, childhood struggles, and heritage, is all part of the skillset expected from mental health providers. In sharing about themselves, participants showed not just what led them to the mental health professions in the first place, but also the values they learned and developed during training: self-awareness, vulnerability, resilience.

These overarching trends can productively be viewed through the related theoretical lenses of communities of practice (CoP) and situated learning theory (SLT). In CoP (35), learning and professionalization are viewed as a collective creation of knowledge. Effective CoPs look to develop creative and practical solutions to common problems encountered in clinical practice (36). These educational collectives are shaped by three main elements: (1) *Mutual engagement* to continue the tradition of learning, practice, or clinical care; (2) *Joint enterprise* toward a common goal (of improving the lives of children and families in this instance); and (3) *Shared repertoire* of actions, interactions, roles, and routines, i.e., the “tools of the trade.” SLT (37) complements and extends CoP by emphasizing groupwide, social-based learning that is embedded into everyday activities, usually in an unintentional rather than a deliberate way. It further highlights the interactions between newcomers and those who are more experienced or knowledgeable (38).

### 4.3. Learning in place: transition, generativity, and legacy

On the cusp of graduating, participants looked forward with excitement (and some inevitable apprehension) to the next phase of their professional and personal development. Each of them was eager to share and to pass on to others what they had learned, experienced, and internalized during training. They wanted their students to be treated and taught as they in turn had been: they wanted to be active in that process. They wanted to pay it forward, not just as clinicians, but as teachers, mentors, and yes, even as role models to others. They were interested in future generations, in the transmission of their values, in generativity.

They were not alone in these aspirations. They belong to a field that values mentorship (39) more than most—perhaps because mentorship can be conceptualized as development occurring across generations, rather than within individuals. Mentorship initiatives in child and adolescent psychiatry have interconnected not just generations, but medical students across the United States (40), and young scholars around the globe (41, 42).

The legacies that participants dreamed ahead to were largely people-based: they involved their students and patients, their own families. But one additional legacy of theirs would remain palpable: their likeness in a large oil painting, hanging permanently at their training institution. How can such a concrete legacy be thought of?

Its site of display will be welcoming and warm, it will be playful and inviting. The rich array of skin tones it includes will project a sense of pride, a message that everyone belongs and contributes to a larger cause (43). It will also help decry and reject long-standing race-based exclusion (44) *Heritage and Legacy* is a product of its time. It served as a point of community-building during the lengthy isolation imposed by the COVID-19 pandemic. And it became a concrete way to confront structurally embedded racism and exclusion in the wake of the on-camera murder of George Floyd in May 2020 and the mass demonstrations that followed nationally. It became a timely response to the University's call to “[r]eview public art and iconography and add works that represent contributions of a broad range of community members and alumni” (45, p. 18). It coincided and indeed epitomized School- and Center-wide initiatives to address diversity, equity, and inclusion as top strategic priorities. The portrait will hang in a prominent, naturally lit wall, displayed across from another oil painting. That portrait, of historical significance, is of the first tenured Black professor at the Yale School of Medicine (46). To this day, it remains the sole such portrait, but hopefully the first of many.

In the final analysis, *Heritage and Legacy* may be most effective and enduring at eliciting questions, as a disruptive agent to ““make strange”—that is, to trouble one's assumptions, perspectives, and ways of being in order to view anew” (47, p. 973).

### 4.4. Limitations

We recognize several limitations. First, even as the painting was inclusive of three professional disciplines in child and adolescent mental health, it does not provide a comprehensive view. On the one hand, this is inevitable given the limited square footage of the canvas. On the other, it is a reminder of the countless other individuals who contribute each day to the overall mission of bringing care to children and their families: educators, nurses, administrative and support staff, janitors and food service staff—and so many others who go routinely unacknowledged and unrecognized. Second, we did not capture the narratives of those trainees who did not participate, especially who passed on the opportunity to sit for the portrait. It could be that their views may have shed other unique perspectives. Third, our interview guide was fairly unstructured; with questions more specific to particular anchors around educational stages and other personal influences, it is possible we could have obtained more granular or different information. Fourth, even as the painting is unique, the themes contained within its narratives may not be. For example, without a comparison to other disciplines, we cannot state that some of the factors driving participants' careers, such as comfort with ambivalence and uncertainty, are unique to the child mental health professions. Finally, participants were able to react to the painting at its state of completion at the time of their sitting; it would have been productive had they been able to react to the final version, whether individually or as a group.

## 5. Conclusion

In closing, the lengthy process of creating an oil canvas depicting a multidisciplinary group proved a powerful vehicle for self-reflection by those portrayed. As professionals dedicated to child, adolescent, and family mental health—to development—participants were well attuned to their own developmental trajectories: the heritage of their upbringing, the process of education and socialization into their professional selves, and their hopes and dreams for the future as they readied to graduate and planned ahead to the legacy they would one day leave behind. A *Legacy* in its own right, the oil painting will hang permanently at the institution where they trained: welcoming, inviting, eliciting—a beacon for future generations of trainees to come.

## Data Availability

The raw data supporting the conclusions of this article will be made available by the authors, without undue reservation.
