# Supplementary material for: Narratives of heritage and legacy: Child and adolescent mental health trainees portrayed
Source: Front Child Adolesc Psychiatry. 2023 Feb 23;2:1104637. doi: 10.3389/frcha.2023.1104637 (PMC11747910; doi:10.3389/frcha.2023.1104637)
Supplement: Supplementary file 1 [file Datasheet1.pdf]

WELCOME TO THE HERITAGE AND LEGACY PROJECT

SENSITIZING QUESTIONS

1. What was it like growing up as the youngest/oldest/middle/only child?
2. What's your favorite family tradition?
3. What does it mean to be vulnerable? How has vulnerability played a role in your journey as an individual?
4. How can someone earn your trust?
5. From the markets in Mumbai to the brownstones in Brooklyn, what location has made the most impact in your life? The most growth?
6. What kind of jobs did you have before a career in Medicine/Psychiatry/mental health? What did you learn from them?
7. Within Medicine/Psychiatry/mental health?
8. How do you keep yourself motivated and interested in your work?
9. When will you know you've "made it"?
10. What is the best advice you have been given?
11. What has been the biggest opposing force you encountered on your life journey so far?
12. What has been your favorite accomplishment?
13. Can you recall a specific moment that gave you a new perspective on life? What lessons did you learn?
14. If you and I were to trade places, tell me one thing about yourself that I should know.
15. What is a lesson that you learned from a child or adolescent?
16. Is there a time when things didn't go the way you wanted--like a promotion you wanted and didn't get, or a project that didn't turn out how you had hoped? How did you deal with that?
17. How do you pick yourself back up after making a mistake at work?
18. What were you like in high school? In grade school, junior high? What stands out?
19. You have clearly achieved a lot in your life so far. Is there anything that you have not done yet or would like to do in the future?
20. When have you been the most content in your life?
21. When was the last time you cried and why?
22. Teach me something about you that I don't know in the next five minutes
